# Optimal Keratoplasty for the Correction of Presbyopia and Hypermetropia

**DOI:** 10.1155/2017/7545687

**Published:** 2017-04-18

**Authors:** Daniele Veritti, Valentina Sarao, Paolo Lanzetta

**Affiliations:** ^1^Istituto Europeo di Microchirurgia Oculare (IEMO), Udine, Italy; ^2^Department of Medicine‐Ophthalmology, University of Udine, Udine, Italy

## Abstract

*Purpose.* To evaluate prospectively the safety and efficacy of optimal keratoplasty for the correction of hyperopia and presbyopia. *Methods*. Consecutive patients undergoing bilateral optimal keratoplasty for refractive presbyopic and hypermetropic corrections were enrolled. Each patient received a complete ophthalmologic examination at baseline, 1 hour, 1 day, 1 week, 1 month, 3 months, and 6 months after treatment. *Results*. The study included 40 consecutive eyes of 20 patients. All patients reached the 6-month follow-up. No serious intra- or postoperative complications were recorded. Monocular and binocular uncorrected near visual acuities improved significantly during the follow-up (*p* < 0.001). Binocular uncorrected distance visual acuity in presbyopic patients improved from 0.28 logMAR to a maximum of 0.04 logMAR (from 20/38 to 20/22 Snellen equivalent) the day after the treatment and remained significantly better than baseline until the end of the follow-up. A significant improvement of patient satisfaction for near (*p* < 0.001) and distance (*p* = 0.007) activities was seen the day after treatment and was maintained throughout the follow-up. *Conclusions.* Optimal keratoplasty is a safe, noninvasive, rapid, pain-free, office-based procedure. It offers low to moderate hyperopes and presbyopes an improvement in uncorrected near visual acuity while maintaining or improving their distance visual acuity.

## 1. Introduction

Presbyopia is the most common ocular condition associated with ageing of the eye. It leads to a progressively impaired ability to focus on near objects, especially in the emmetropic or hyperopic eyes.

In the recent years, a series of treatments have been proposed to avoid the use of reading glasses. Among them, conductive keratoplasty (CK), laser thermal keratoplasty (LTK), and multifocal laser in situ keratomileusis (LASIK) are those most used in clinical practice [1–4]. The advancements in the design of multifocal intraocular lenses have made these a valuable option too [5]. More recently, optimal keratoplasty (Opti-K, NTK Enterprises, Inc.) has been proposed as a new office-based laser vision correction procedure performed with a continuous-wave thulium fiber laser that reshapes the cornea to temporarily improve the symptoms of presbyopia and correct low to moderate hyperopia similarly to LTK. Differently from LTK, in Opti-K, laser light is transmitted through a sapphire application window, which reduces the temperature at the epithelial level below the threshold of thermal damage. Nevertheless, the rise of temperature within the anterior stroma is adequate to cause corneal shape change compacting the anterior stromal lamellae. The application of such epithelium and basement membrane-sparing strategy avoids the pitfalls of LTK and CK [6]. These procedures produced effective refractive treatment but were associated with side effects due to epithelium/basement membrane damage [7, 8].

Opti-K produces 16 spots with a diameter of 500 *μ*m at 3.0 and 3.6 mm from the optical center. Indeed, a minimalist treatment is required to provide significant corneal shape change with Opti-K. At the 4 mm optical zone, the sagittal displacement typically required to produce a diopter (D) change is 5.6 *μ*m. Topographically, optimal keratoplasty produces a rosette-shaped pattern of alternating steeper and flatter sectors resulting in a sort of multifocal cornea [9].

The purpose of the present study is to evaluate the safety and the efficacy of optimal keratoplasty treatment for correcting low to moderate hyperopia and for improving uncorrected near visual acuity.

## 2. Methods

This safety and effectiveness study is a prospective, single-arm, nonrandomized, unmasked clinical trial. This study follows the tenets of the Declaration of Helsinki and was approved by the local institutional review board.

### 2.1. Patient Population and Examinations

Consecutive patients were enrolled and underwent bilateral optimal keratoplasty for refractive presbyopic and hypermetropic corrections.

The inclusion criteria were (a) patient was at least 40 years of age, (b) low to moderate hypermetropia (manifest refraction: sphere between +1 and +2.5 D, absolute cylinder ≤ 1 D) or presbyopia (with presbyopic adds between +1 D and +2.75 D), (c) documented stable refraction, and (d) corrected distance visual acuity (CDVA) of 33 Early Treatment Diabetic Retinopathy Study (ETDRS) letters or better. The exclusion criteria were (a) nystagmus, (b) corneal diameter ≤ 9 mm, (c) central corneal thickness ≤ 500 *μ*m, (d) dry eye disease, (e) severe blepharitis, and (f) residual, recurrent, or active corneal disease or abnormality.

Baseline and follow-up examinations included measurement of uncorrected distance visual acuity (UDVA), uncorrected near visual acuity (UNVA), CDVA, manifest refraction, corrected near visual acuity (CNVA), presbyopic add, corneal optical coherence tomography (OCT), corneal pachymetry, slit lamp examination of the anterior segment, and dilated fundus examination.

Distance and near visual acuities were measured with ETDRS charts and standardized procedures. Visual acuities are reported in logMAR. Patient-reported outcomes were measured using a visual analog scale with regard to UDVA satisfaction, UNVA satisfaction, and global satisfaction.

All the eyes were evaluated at baseline, 1 hour, 1 day, 1 week, 1 month, 3, and 6 months after treatment.

### 2.2. Device Description and Treatment Plan

**Figure 1:**
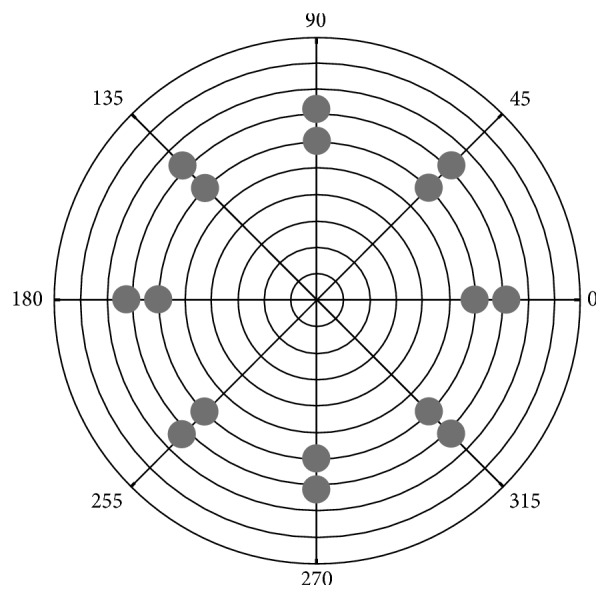
Pattern of the output laser beam. Inner and outer spot rings are applied at 3 and 3.6 mm from the center.

The device used in the present study is the NTK Optimal Keratoplasty (Opti-K) System. It is a continuous-wave thulium fiber laser device for irradiation of corneal tissue. Output beam is directed onto the cornea in a ring pattern (6 and 7.2 mm diameter) (Figure 1). Patient is lying in supine position, then the corneal epithelium is protected from thermal damage with a sapphire application window/suction ring. Thereafter, laser is applied at 1.93 *μ*m wavelength. The laser is typically operated with a total delivered power of 0.80–1.28 W for a period of 150 ms. The duration of the entire procedure is approximately 10–15 minutes. Retreatment was allowed at investigator's discretion based on measurement of UNVA and UDVA.

### 2.3. Data Analysis

The primary efficacy endpoint is mean UNVA change at 6 months. Secondary efficacy endpoints included UDVA in the hypemetropic/presbyopic eyes. Safety assessments include a tabulation of complications, adverse events, and patient symptoms and UCVA in the pure presbyopic eyes. Efficacy endpoints were analysed using a generalized estimating equation model to account for intersubject correlation. Serial comparisons of pretreatment and posttreatment outcomes were performed using paired *t*-test or Wilcoxon matched pairs nonparametric test according to the Gaussianity or non-Gaussianity of the distributions. In serial comparisons, the null hypothesis was rejected for *p* values <0.05.

## 3. Results

Forty eyes of 20 patients were included in the study. Patient received a mean (±SD) of 1.4 ± 0.5 treatments during the study period. Sixteen eyes (8 patients) required an additional treatment during the follow-up. Retreatment was performed 45 days after the primary treatment, on average. Both the single- and double-treated eyes were evaluated before primary treatment (baseline evaluation) and were then followed up for the study period (6 months). Baseline characteristics are reported in Table 1.

### 3.1. Safety

No serious adverse events or complications, no permanent loss of ≥1 line of UDVA or UDVA < 20/40, and no induced astigmatism ≥ 1 D were recorded. Two patients (5%) complained of temporary glare that was resolved within one week. No change of more than 0.1 logMAR in best-corrected distance visual acuity was recorded during the follow-up.

### 3.2. Visual Acuities

**Figure 2:**
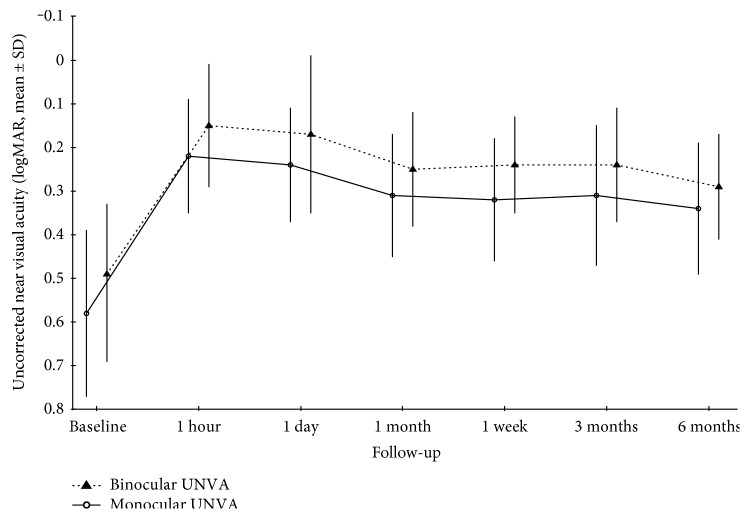
Monocular and binocular uncorrected near visual acuity changes during the follow-up.

**Figure 3:**
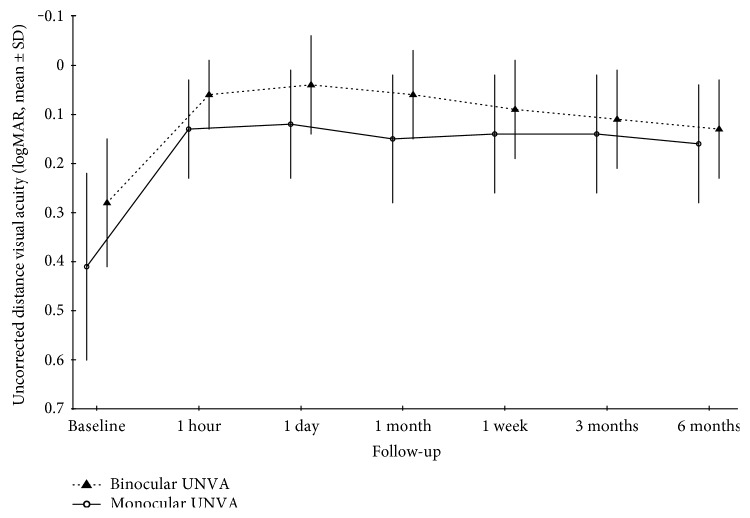
Monocular and binocular uncorrected distance visual acuity changes in hypermetropic/presbyopic patients.

Monocular and binocular UNVA improved significantly during the follow-up (*p* < 0.001) (Figure 2 and Table 2). Binocular UDVA in presbyopic patients improved from 0.28 to a maximum of 0.04 logMAR (from 20/38 to 20/22 Snellen equivalent) the day after the treatment and remained significantly better than baseline until the end of the follow-up (Figure 3 and Table 2).

### 3.3. Patient-Reported Outcomes

A significant improvement of patient satisfaction for near (*p* < 0.001) and distance (*p* = 0.007) activities was seen the day after treatment and was maintained throughout the follow-up (Table 2).

### 3.4. OCT Changes

**Figure 4:**
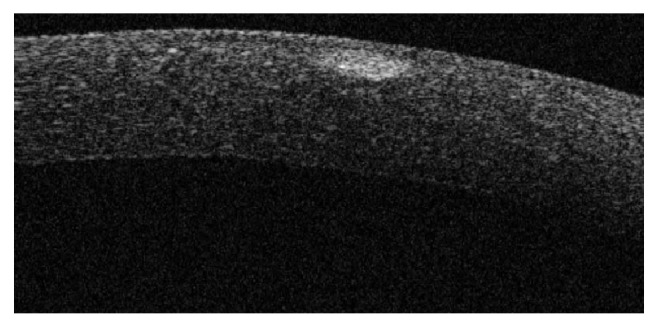
Corneal OCT reveals a hyper-reflective area immediately behind the epithelial layer in a 50-year-old patient one hour after undergoing optimal keratoplasty.

Corneal OCT allowed to identify compacted lamellae as hyper-reflective areas immediately behind the epithelial layer and extending up to 100 *μ*m into the stromal layer (Figure 4). This finding is noticeable soon after treatment and gradually fades during the follow-up. Median time to disappearance is 5.4 months. In 45% of the eyes, hyper-reflective areas were still noticeable at the end of the follow-up.

One hour after treatment, no corneal epithelial defects were observed. In one patient (2 eyes, 5%), corneal OCT revealed a thickening of the epithelial layer.

## 4. Discussion

A growing demand for effective treatment of presbyopia is impacting the ophthalmology practices. Numerous surgical and laser procedures to correct presbyopia and hyperopia have been tested over the past decade, whose main issues being unpredictable visual outcomes, irreversibility, regression, and corneal damage. In 2002 and in 2004, CK has been reported to be effective for the treatment of low to moderate hyperopia and presbyopia [1, 2]. CK and LTK efficacy lies in the fact that heat coagulation of the corneal stroma alters the corneal curvature. Thermal alteration of corneal collagen occurs when the tissue is exposed to temperatures of 58° to 75°C. In detail, the collagen matrix changes from the triple helix formation to a partially coiled structure resulting in shrinkage of collagen fibers [10–14]. Opti-K, with the use of sapphire applanation ring, confines thermal elevation into the anterior corneal stroma, which produces limited thermally damaged zones that compact the anterior stromal lamellae while preserving the overlying epithelium. This is in stark contrast to antecedent technologies like CK and LTK which both damage the epithelium and the basement membrane causing discomfort and fibrotic wound-healing response.

Optimal keratoplasty, similarly to CK, produces multiple conoids of Sturm generating useful corneal multifocality [15]. Despite the presence of multiple intervals of Sturm, patients did not report blur or decreased vision quality. This may be imputable to neuro-optical phenomenon for blur suppression and neuro-adaptation. The present is the first prospective and peer-reviewed validation of optimal keratoplasty. We reported a significant improvement in UNVA. UDVA improved in hyperopic patients and was maintained in emmetropic patients. The mean effect duration after the first treatment was 45 days. The peak of efficacy was seen during the first weeks of the follow-up, and UDVA and UNVA then progressively decline between months 3 and 6. But, a statistically significant visual benefit over baseline values was maintained throughout the follow-up. Longer follow-ups are indeed needed to quantify the regression of the effect and the efficacy of multiple retreatments. In the present study, no specific intra- or postoperative complications were recorded. Quality of vision, as evaluated by the patients, was high and stable. In all cases, the postlaser recovery was immediate. All patients returned to their normal activity the following day. Most patients that required a repeated treatment presented with a self-reported decline of visual benefit asking for undergoing a repeated procedure.

## 5. Conclusion

In conclusion, optimal keratoplasty is a safe, noninvasive, rapid, pain-free, office-based procedure. It offers low to moderate hyperopes and presbyopes a temporary improvement in uncorrected near visual acuity while maintaining or improving their distance visual acuity. Further studies, with longer follow-up and multiple repeated treatments, are warranted to evaluate long-term safety.

## Figures and Tables

**Table 1:** Baseline characteristics.

Demographics		Value
Age, years mean (±SD)		55 (±12)
Female sex, *n* (%)		13 (65%)
Hypermetropic/presbyopic eyes, *n* (%)		24 (60%)
Pure presbyopic eyes, *n* (%)		16 (40%)
Central corneal thickness, *μ*m mean (±SD)		561 (±21)
Uncorrected monocular near visual acuity, logMAR mean ± SD (Snellen)		0.58 ± 0.19 (20/76)
Uncorrected binocular near visual acuity, logMAR mean ± SD (Snellen)		0.49 ± 0.16 (20/62)
Uncorrected monocular distance visual acuity, logMAR mean ± SD (Snellen)	Hypermetropic/presbyopic eyes	0.41 ± 0.19 (20/51)
	Pure presbyopic eyes	0.04 ± 0.05 (20/22)
Uncorrected binocular distance visual acuity, logMAR mean ± SD (Snellen)	Hypermetropic/presbyopic eyes	0.28 ± 0.13 (20/38)
	Pure presbyopic eyes	0 ± 0.02 (20/20)
Astigmatism, diopters, mean ± SD		0.67 ± 0.55
Near add, diopters, mean ± SD		2.01 ± 0.38

SD: standard deviation; *n*: number.

**Table 2:** Visual acuity outcomes.

		Baseline	1 hour	1 day	1 week	1 month	3 months	6 months	Significance
All eyes	Monocular UNVA logMAR ± SD (Snellen)	0.58 ± 0.19 (20/76)	0.22 ± 0.13 (20/33)	0.24 ± 0.13 (20/35)	0.31 ± 0.14 (20/41)	0.32 ± 0.14 (20/42)	0.31 ± 0.16 (20/41)	0.34 ± 0.15 (20/44)	
	*p* value		*p* < 0.001	*p* < 0.001	*p* < 0.001	*p* < 0.01	*p* < 0.01	*p* < 0.01	*p* < 0.001
	Binocular UNVA logMAR ± SD (Snellen)	0.49 ± 0.16 (20/62)	0.15 ± 0.1 (20/28)	0.17 ± 0.11 (20/30)	0.25 ± 0.13 (20/36)	0.24 ± 0.11 (20/35)	0.24 ± 0.13 (20/35)	0.29 ± 0.12 (20/39)	
	*p* value		*p* < 0.01	*p* < 0.01	*p* < 0.01	*p* < 0.01	*p* < 0.01	*p* < 0.01	*p* < 0.001

Double-treated eyes	Monocular UNVA logMAR ± SD (Snellen)	0.60 ± 0.21 (20/80)	0.30 ± 0.21 (20/40)	0.27 ± 0.20 (20/37)	0.34 ± 0.16 (20/44)	0.39 ± 0.17 (20/49)	0.30 ± 0.18 (20/40)	0.32 ± 0.17 (20/42)	
	*p* value		*p* < 0.01	*p* < 0.01	*p* < 0.01	*p* < 0.05	*p* < 0.01	*p* < 0.05	*p* = 0.008
	Binocular UNVA logMAR ± SD (Snellen)	0.51 ± 0.22 (20/65)	0.17 ± 0.15 (20/30)	0.19 ± 0.15 (20/31)	0.28 ± 0.15 (20/38)	0.30 ± 0.17 (20/40)	0.25 ± 0.16 (20/36)	0.28 ± 0.17 (20/38)	
	*p* value		*p* < 0.01	*p* < 0.01	*p* < 0.01	*p* < 0.05	*p* < 0.01	*p* < 0.01	*p* = 0.012

Hypermetropic/presbyopic eyes	Monocular UDVA logMAR ± SD (Snellen)	0.41 ± 0.19 (20/51)	0.13 ± 0.1 (20/27)	0.12 ± 0.11 (20/26)	0.15 ± 0.13 (20/28)	0.14 ± 0.12 (20/28)	0.14 ± 0.12 (20/28)	0.16 ± 0.12 (20/29)	
	*p* value		*p* < 0.01	*p* < 0.01	*p* < 0.01	*p* < 0.01	*p* < 0.01	*p* < 0.01	*p* < 0.001
	Binocular UDVA logMAR ± SD (Snellen)	0.28 ± 0.13 (20/38)	0.06 ± 0.07 (20/23)	0.04 ± 0.1 (20/22)	0.06 ± 0.09 (20/23)	0.09 ± 0.1 (20/25)	0.11 ± 0.1 (20/26)	0.13 ± 0.1 (20/27)	
	*p* value		*p* < 0.01	*p* < 0.01	*p* < 0.01	*p* < 0.01	*p* < 0.01	*p* < 0.01	*p* < 0.001

Pure presbyopic eyes	Monocular UDVA logMAR ± SD (Snellen)	0.04 ± 0.02 (20/22)	0 ± 0.04 (20/20)	0.03 ± 0.06 (20/21)	0.04 ± 0.1 (20/22)	0 ± 0.05 (20/20)	0 ± 0.04 (20/20)	0.03 ± 0.06 (20/21)	
	*p* value		*p* > 0.05	*p* > 0.05	*p* > 0.05	*p* > 0.05	*p* > 0.05	*p* > 0.05	*p* > 0.05
	Binocular UDVA logMAR ± SD (Snellen)	0 ± 0.05 (20/20)	−0.04 ± 0.06 (20/20)	−0.03 ± 0.05 (20/20)	0 ± 0.1 (20/20)	−0.06 ± 0.06 (20/18)	−0.05 ± 0.05 (20/18)	−0.04 ± 0.06 (20/20)	
	*p* value		*p* > 0.05	*p* > 0.05	*p* > 0.05	*p* > 0.05	*p* > 0.05	*p* > 0.05	*p* > 0.05

Patient satisfaction for near activities		33 ± 20	—	76 ± 20	71 ± 20	75 ± 19	68 ± 16	71 ± 16	
	*p* value			*p* < 0.001	*p* < 0.001	*p* < 0.001	*p* < 0.01	*p* < 0.05	*p* < 0.001

Patient satisfaction for distance activities		70 ± 23	—	82 ± 10	82 ± 14	84 ± 14	87 ± 11	84 ± 14	
	*p* value			*p* < 0.05	*p* < 0.05	*p* < 0.01	*p* < 0.01	*p* < 0.05	*p* = 0.007

UNVA: uncorrected near visual acuity; UDVA: uncorrected distance visual acuity.
